# Occupational burnout and chronic fatigue in the work of academic teachers–moderating role of selected health behaviours

**DOI:** 10.1371/journal.pone.0280080

**Published:** 2023-01-26

**Authors:** Agnieszka Springer, Karolina Oleksa-Marewska, Agata Basińska-Zych, Iwona Werner, Sylwester Białowąs

**Affiliations:** 1 Department of Finance and Banking, WSB University in Poznan, Poznan, Poland; 2 Department of Market Research and Services Management, Poznan University of Economics and Business, Poznan, Poland; North South University, BANGLADESH

## Abstract

Increasing and changing demands for academic teachers worldwide are leading to different consequences, some of which are negative, such as physical and mental health impairment. With the job demands-resources model and the transactional model of occupational stress adopted as a theoretical framework, a cross-sectional study among Polish academic teachers was conducted. The aim of the study is to identify the role of vital personal resources understood as selected health-promoting behaviours, such as (1) stress-coping strategies, (2) sleep hygiene, and (3) using annual vacation leave, in the process of the regeneration. In a cross-sectional survey, the following variables have been assessed: 1) work-related stress using the Psychosocial Risk Scale, 2) occupational burnout using the Oldenburg Burnout Inventory (OLBI), 3) chronic fatigue using the Polish adaptation of the Checklist Individual Strength (CIS), and selected health behaviours of academic teachers 4) stress-coping strategies with the help of the Polish version of Mini-COPE and 5) rest: the amount of sleep and vacation days devoted to rest using a short questionnaire designed by the authors. The sample (N = 340) was comprised of academic teachers employed at Polish higher education institutions who have experienced changes in the work environment in recent years. We conducted a multiple regression analysis to determine the relationships among stress, burnout, and chronic fatigue, looking at coping strategies and rest as a moderator. The results indicate that there is a strong relationship between stress resulting from an excessively demanding work environment on the one hand and occupational burnout and chronic fatigue on the other. At the same time, selected health behaviours of academic teachers only slightly moderate the analyzed relationship. Avoidance strategies strengthen the relationship between stress and its negative consequences, while rest and—to a limited extent—the amount of sleep only slightly contribute to weakening the analyzed relationship.

## Introduction

Recent decades have brought numerous changes in the academic environment [1,2], which affect academic teachers in different countries [3–5]. Due to transformation of political and socio-economic trends universities have shifted from traditional academic values into managerial logics [2,6], thus providing many changes in the working conditions for their employees. Among their biggest professional challenges, academic teachers list giving classes while being engaged in research work, self-development (gaining new knowledge and qualifications), and performing administrative and academic duties resulting from being employed at a given unit [7,8]. Academics’ work has become more intensive and their role and tasks more diversified, while their research freedom and employment stability has been reduced, and in the social dimension, competition for limited resources has increased–all of which raises the stress levels of academic teachers [9]. As the growing interest in employees’ wellbeing can be observed, the sample of academic teachers hasn’t been comprehensively investigated. Studies identify some specific psychosocial stressors in academics working conditions, like work overload, time constraints and extended working hours [10,11], lack of research finance, role insufficiency, role overload [3,12], “public or perish” pressure [13], decreasing in autonomy and control [14], increasing in students’ number [12], the interaction of Internet-based education [15], job insecurity, excessive administrative work [3,14], role ambiguity or poor faculty communication [10]. Moreover, at least in the last two decades, the stress indicators increased alarmingly in this occupational group across the globe [3,12,16] elevating the impact of negative outcomes of the stress: lower levels of satisfaction and well-being, impaired health and lower productivity [17].

Similarly, like at global scale, the universities in the eastern Europe are currently experiencing significant changes which evoke the need to continuously adapt to external demands [18]. Thus, eastern Europe academics face similar challenges which are bounded with increasing the competitiveness of universities and dynamic rise in the number of students [2,19] and specifically in Poland academics are experiencing the effects of major reforms in the higher education sector [18,20,21].

In the face of constant external changes resulting in a sense of uncertainty and growing demands on the work of academic teachers, the question arises about the possibility of preventing negative outcomes in the area of employee well-being [9,22]. Due to the limited possibilities in terms of reducing the demands of the work environment and taking into account the fact that little is known about the importance of individual factors regulating the experience of occupational stress in teachers group [23], the question was posed about the role of selected personal resources in the coping with work requirements. The focus was on one of the categories of personal resources, namely health-promoting behaviours, which play an important role in regaining vitality and improving employee well-being [24]. The above considerations fit into two theoretical concepts: job demands-resources (JD-R) [25] model and the transactional model of occupational stress developed by Cox [26]. First studies devoted to the JD-R model, which is currently one of the most popular tools among organisational psychologists, were published at the beginning of the 21^st^ century [25,27], but it is still being developed and refined [28,29]. Integration of different concepts allowed the authors to create an universal approach to diagnosing employee wellbeing, which can be used regardless of the profile of organisation and the type of job, also in the academic environment [30].

The basic tenet of the JD-R model is that each job comes with specific stress-related risk factors which can be divided into two categories: job demands and job resources. Job demands refer to physical, psychological, social, or organizational aspects of the job that require durable physical / psychological effort and also lead to certain physiological and psychological costs [31]. Job demands can therefore be identified with psychosocial hazards associated with job content as described by Cox [32,33]. What is crucial for both the JD-R model and the theory developed by Cox and associates are employees’ subjective perceptions of the situation at work as potentially harmful to their physical or mental state.

At the same time, in the JD-R it is assumed that the negative consequences of excessive demands can be moderated to some extent by providing the worker with the necessary resources. The relationship between job demands and resources initiates two independent processes. The health impairment process results from high job demands and leads to negative consequences for workers’ health and state of mind [34]. The motivation process, emerging from the availability of resources, leads to increased engagement and job satisfaction. In every work environment, including higher education institutions, it is therefore necessary to provide workers with the tools required to improve these resources.

Both the JD-R model and the analogous transactional model of psychosocial hazards allow researchers to assess the wellbeing of workers in terms of health and motivation. High job demands and the unavailability of resources, both personal and organizational, are sources of stress associated with job content and job context, which can lead to negative consequences for motivation and health. The analysis carried out by the authors focuses on two consequences of a stressful work environment: occupational burnout and chronic fatigue. While the former has been widely analyzed in organizational psychology literature [35,36], the latter is a term more often found in medicine [37,38], although it is also indicated as a negative consequence of excessive job demands in the JD-R model [25]. Chronic fatigue is a concept strongly linked to occupational burnout; besides the aforementioned JD-R model, it is also found in the conservation of resources (COR) model as a result of difficulties in adhering to one’s values in the workplace [39]. In medical terminology, Chronic Fatigue Syndrome/Myalgic Encephalomyelitis (CFS/ME) is understood as a long-term illness, characterized by extreme, disabling, persistent unexplained fatigue that is not due to exertion and not relieved by rest [38].

According to the duration, fatigue is classified as acute (lasting less than one month), prolonged (from one month to six months), and chronic fatigue (more than six months). Acute fatigue typically decreases after a rest or health recoverywhile uncontrolled prolonged and chronic fatigue can be limited by the physical and social activities [40]. Nevertheless, if the effort is excessive, the rest is inadequate (too short or under inappropriate conditions), or other factors that may have a negative influence on well-being occur (deterioration of health, social situation, etc.), chronic fatigue develops. Chronic fatigue may also be a consequence of high occupational stress or occupational burnout [37,41] or, on the contrary, working without intellectual stimulus, with a low level of challenges and motivators [42]. Chronic fatigue can cause temporary or permanent inability to work, it may deteriorate the quality of life, as well as the effectiveness and safety at work [43]. What is worth mentioning, chronic fatigue may not only be a consequence of overcoming difficulties but also a cause ofa disease. The spectrum of symptoms related to chronic fatigue is very extensive. Most often fatigue affects the emotional sphere and is manifested in reluctance to work, irritability, depression, sometimes aggressiveness or apathy, sleep disturbances, and and dysregualtionof vegetative functions [40]. Individuals who suffer from chronic fatigue are characterized by the ineffective actions, reluctance to engage, reduced motivation, decreased concentration, and experiencing negative emotions [43]. Therefore, diagnosing chronic fatigue requires a multi-faceted perspective.

Interdisciplinary literature is taking an increased interest in chronic fatigue, perceiving it as an important work-related personal factor which can affect a worker’s health, job attitude, efficiency, and workplace safety [44]. It is indicated that, in the case of both occupational burnout and chronic fatigue, the main predictor is work-related stress. Although both concepts are strongly connected and possess a common component related to physical exhaustion, occupational burnout is more strongly linked with motivational consequences, while chronic fatigue is linked with health consequences. However, both concepts can lead to a decrease in employee productivity, which has wide economic and social implications [39].

Among negative consequences related to academics’ work and its psychosocial and physical hazards one indicates burnout [4,45] and chronic fatigue syndrome [46]. Academics can experience higher burnout while perceiving the organizational culture as unsupportive [47] and the employment climate as highly demanding [48], which if not balanced with an increase in organizational resources, thereby becoming a challenge for both individuals and organizations [49]. The main causes of occupational burnout of academic teachers include stress resulting from work overload and from spending too much time on work duties [3,8,50] and similar factors connected to extensive job duties are identified as causes of chronic fatigue among teachers [46]. All this problems are actual in the academic life in Poland both contemporary quantitative [51] and qualitative [52] research indicates problem with occupational burnout and signal some other challenges for the future, like difficulties at concentration at work and conducting creative research [52].

It was pointed out that burnout and chronic fatigue syndrome experienced by academics are two separate and distinguishable constructs, although they both have characteristic of fatigue in common [46]. Nevertheless, there are some other common factors between chronic fatigue syndrome and burnout connected to health issues, such as headaches, sleep disturbances and exhaustion [53,54] thus suggesting probable important role of physical and psychological recovery for the prevention of chronic fatigue syndrome and burnout.

### Research hypothesis

Although for over a dozen years within the JD-R model the importance of personal resources in effective handling the organizational requirements has been indicated [36,55,56], the need to conduct further research in this area is necessary [29]. While the positive role of optimism and self-efficacy or intrinsic motivation as important personal resources has been confirmed [55,57–61], the evidence regarding the role of health-promoting behaviours in coping with job requirements is insufficient.

Lack of studies that include health behaviours in the area of the JD-R, and the growing need for the implementation of effective health-promotion programs [62] point out that health-promoting behaviours can play an important role in the health and motivation process. Health promoting programs are very varied and may take the form of behaviours aimed at maintaining or restoring one’s health (health-promoting behaviours) or behaviors which cause damage to one’s health now or in the future (health-impairing behaviours) [24]. Although a significant scientific research of the past decades proves the positive effects of health practices such as sufficient restorative sleep, exercise and regular and healthy meals on physical and mental health improvement, regardless of age, gender and economic status [37,63–65] research on the role of health-related behaviours in the prevention of burnout and chronic fatigue of professional group of academic teachers are scarce.

Being aware of the diversity of health behaviorus, the article focuses on 3 of them such as: (1) stress-coping strategies, (2) sleep hygiene, and (3) using annual vacation leave.

The assumption that coping strategies may constitute an important personal resource allowing for better coping with requirements is based on findings resulting from the transactional theory of stress. Literature provides different typologies of stress-coping strategies [66–68]. The best known typology developed by Lazarus distinguishes two basic groups of strategies: those focused on active problem-solving and those focused on regulating the experienced emotions. The aim of all strategies alike is to reduce negative emotions [69]. Parkerand Endler (1992), who considered the above typology to be incomplete, added to it a third form of behaviour in a stressful situation, namely avoidance [70]. Although the effectiveness of the particular strategies has not been unambiguously evaluated [71], numerous works suggest that the real problem-solving capabilities of an individual are first and foremost determined by active problem-solving strategies, which also translate into healthy lifestyles and the reduced impact of the negative consequences of stress. This mechanism has also been observed in the area of professional functioning, including the importance of exhaustion as a result of workoverload [72]. Taking this into account, the following hypothesis was formulated:

#### Hypothesis 1

Using active and emotion-centered strategies weakens the relationship between stress and its negative consequences in the form of occupational burnout and chronic fatigue.

Studies on the effectiveness of the different strategies show that avoidance strategies bring the opposite results, contributing to increased negative consequences related to human activity [70,73–75]. Avoidance strategies suppress emotions and divert attention from the sources of stress, which results in the incompatibility of goals and motivational hesitancy in the motivational process [76]. A second hypothesis was therefore formulated:

#### Hypothesis 2

Using avoidance strategies strengthens the relationship between stress and its negative consequences in the form of occupational burnout and chronic fatigue.

Another health-promoting practice is focused on getting enough rest, including sleep. Regular, quality sleep is a condition of maintaining physical and mental health [64,77] and plays a crucial role in maintaining efficiency and providing energy to the organism [78]. Sleep deprivation, in turn, impairs memory [79], the ability to focus, and logical thinking [80]. Behaviours related to the sleep process, which can affect both sleep quality and duration, are collectively called sleep hygiene [81].

According to the Meijman’s and Mulder’s effort-recovery model [82], managing work and non-work demands takes considerable energy that needs to be restored at the end of the day. Research has shown that recovery activities, such as psychological detachment and relaxation, help increase work engagement [63,83,84] and decrease burnout [85]. Based on these studies and the fact that academic teachers often work at night and can thus suffer from sleep deprivation, the third hypothesis was formulated:

#### Hypothesis 3

The amount of sleep moderates the relationship between stress and its negative consequences in the form of occupational burnout and chronic fatigue.

Rest enjoyed during vacation leave, in terms of both its duration and quality, also plays an important role in the process of regenerating the vital resources of academic teachers. In regard to the duration of rest, vacation can be understood as work-free periods lasting for days or even weeks, whereas breaks are usually work-free intervals lasting for minutes or hours at most [86]. Strauss-Blasche et al. (2002) [87] demonstrated that rest not only buffers the effect of occupational and domestic stress on employee wellbeing, but also that being subjected to an excessive workload immediately after coming back from vacation eliminates its positive effects. A positive, though short-lived, effect of vacation on the health and wellbeing of workers was observed by De Bloom et al. [88,89]. Assuming that vacation leave plays an important role in the regeneration of personal resources, a fourth hypothesis was formulated:

#### Hypothesis 4

The number of vacation days devoted to rest moderates the relationship between stress and its negative consequences in the form of occupational burnout and chronic fatigue.

**Fig 1:**
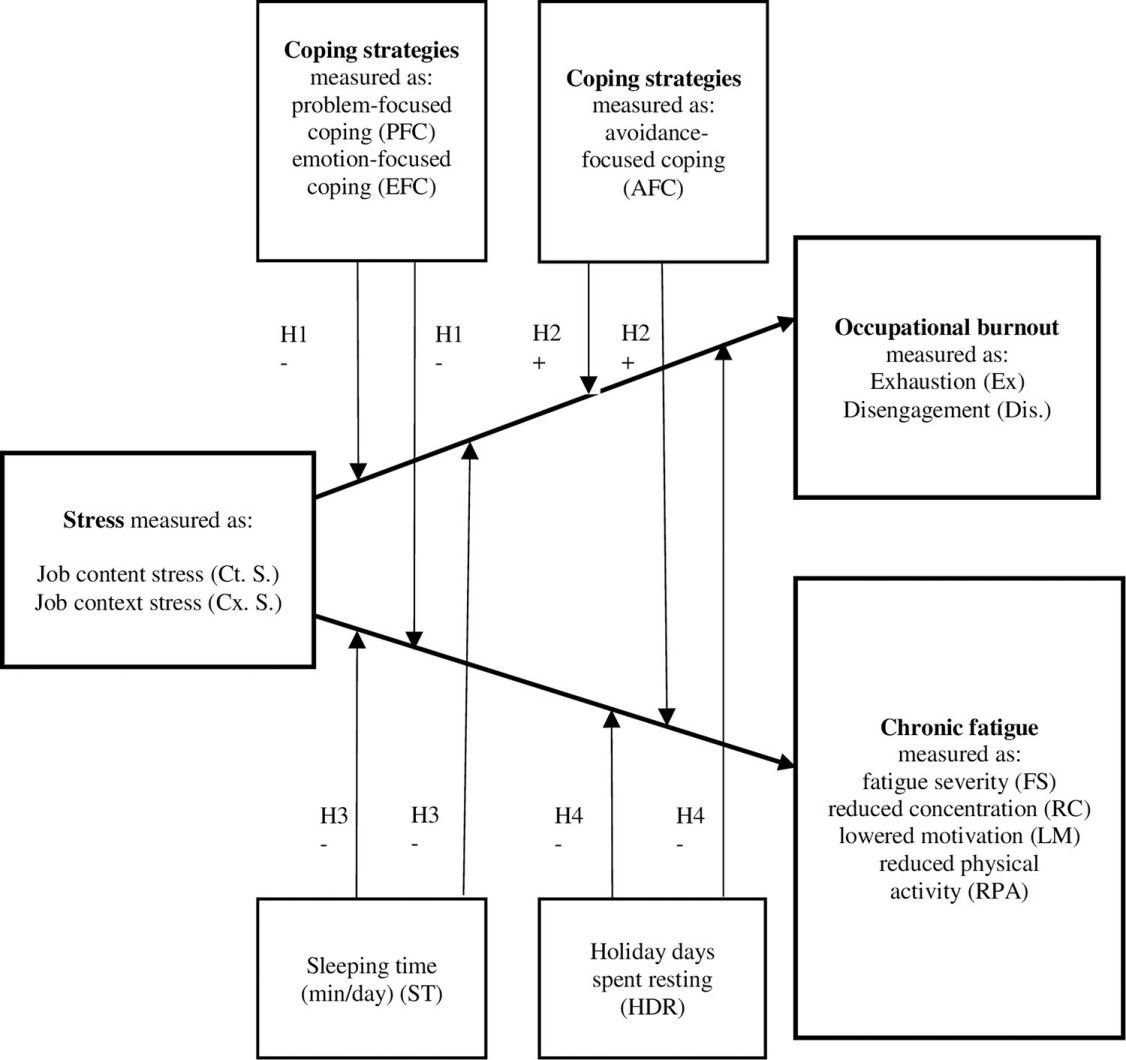
Conceptual model of the moderating role of selected health behaviours on the relationship between stress, occupational burnout, and chronic fatigue.

The conceptual model of the moderating role of selected health behaviours on the relationship between stress, occupational burnout, and chronic fatigue which summarise the hypothesis developed by authors is presented in Fig 1.

## Materials and methods

### Participants and procedure

In order to verify the hypotheses, a quantity survey was conducted among a group of Polish academic teachers. Today, the work environment of Polish academic teachers is shaped by two major higher education reforms, the second of which was introduced in 2018, which mainly changes the quality of academic work by shifting the focus to highly accredited and internationally relevant achievements. The study took place in December 2017 and covered higher education institutions from throughout the country. In 2017 there were 59 public higher education institutions and 239 non-public higher education institutions in Poland [90], with 88% of all academic teachers employed in the first group. Since there was no possibility to collect random sample we have decided for non-random combined sampling. The expected sample size was assumed at the level of at least 300 based on the recommendations of previous scientific works [91,92]. In the first level of sampling two groups were distinguished–the public and the private sector. In the next step twelve public universities were randomly selected. The invitations to participate in the research were sent to all academics of these institutions through their email addresses found on universities’ websites.

Regarding the fact that private universities in Poland usually do not post information about the e-mail addresses of their employees, the respondents from this group were invited using the snowball sampling technique.

In total, about 1200 invitations to participate in the study were sent out and 340 correctly completed questionnaires were obtained. The return rate was 28%. The structure of obtained sample follows the structure of population in terms of sector (public/private) and the degree (MA/PhD/professor).

The survey was conducted online and comprised of an imprint and five questionnaires. The academic teachers were asked to complete self-assessment questionnaires, no sensitive data was collected. The study participants were notified about the purpose of the research before it started. Before filling out the survey, the informed consent was acquired from the participants by using the click-if-you-agree type of online form. The respondents voluntarily participated in this study, their anonymity and confidentiality of information were ensured, and they had the right to withdraw from the study at any time. All obtained data was completely confidential and special codes were used to ensure anonymity. The data was only used for the research purpose. There was no access to data by individuals or organizations other than the investigators. In accordance with institutional requirements of the WSB University in Poznań all research procedures involving human participants used in the study were in accordance with the ethical standards resulting from the Code of Ethics in Science of the WSB University in Poznań (Regulation No. 5/2008 of the Rector of the WSB University in Poznań from 10 April 2008) and the ethical standards of Helsinki Declaration as well as the International Code of Market Research, Opinion and Social Research and ESOMAR Data Analysis in terms of primary data collection, data protection and privacy, publication of results and obligations to the scientist profession.

### Measures

#### Job content and job context stress

The first questionnaire, the orginal version of Psychosocial Risk Scale [93], was used to evaluate work-related stress: the number of risk factors present in the work environment as well as the degree of stressfulness. In the analyses presented in this paper, only the second factor was considered. The questionnaire was developed by Polish researchers Mościcka-Teske and Potocka (2014), based on the concept of T. Cox and verified on a sample of 7623 respondents working in various industries. To assess the psychometric characteristics of the questionnaire, the authors used theoretical validity analysis, discriminant power analysis, factor analysis and internal consistency [89]. The questionnaire is comprised of three independent scales: (1) work content, (2) work context and (3) pathologies. Two aspects of the work environment were used in the analyses, namely work content and work context, which consider Cox’s transactional model of occupational stress [94]. In the analysis, Cronbach’s alpha coefficients for these two scales were 0.92 and 0.99, respectively. Work content includes 14 items and work context includes 28. In all the questions, respondents assessed the degree to which a given factor is stressful to them on a scale from 1 to 3. As suggested by the authors of the questionnaire, the score should be interpreted as follows: for the work content result between 1–1.89 points shows low level of stress; 1.9 to 2.56 -medium, 2.57 to 3 –high, and for job content: 1–1.88 indicates the low level of stress; 1.89 to 2.35- medium, 2.35 to 3 -high [89].

#### Occupational burnout

The second tool was the Oldenburg Burnout Inventory (OLBI), developed by Demerouti et al. (2001) to measure the consequences of the relationship between job demands and resources in the form of occupational burnout on two scales: exhaustion (α = 0.85) and disengagement (α = 0.68). The validity of OLBI has been confirmed in many different studies [95]. Furthermore, in the Polish adaptation of the questionnaire, its reliability and validity were confirmed [96].

#### Chronic fatigue

The third questionnaire was the Polish adaptation [43] of the Checklist Individual Strength (CIS) [97] that served to determine the level of chronic fatigue. This multidimensional questionnaire contains 20 items, which enable to assess the chronic fatigue on the 7-point Lickert scale. The questionnaire allowed the researchers to measure four aspects of chronic fatigue: the severity of fatigue (α = 0.88) lower motivation (α = 0.56), reduced physical activity (α = 0.74), and reduced concentration (α = 0.73). The CIS questionnaire allows for assessing the general level of chronic fatigue, being the sum of the scores of all items and the mean values of each component. The higher the general score, the greater level of chronic fatigue [43]. The authors propose to intepret the general level of chronic fatigue as follows: up to 39 points as low, 40–82 points as medium, and more than 82 as high [43]. The validity of the CIS questionnaire was determined among the working population [97] and is perceived as a good predictor of chronic fatigue [98]. The authors of the Polish adaptation obtained satisfactory results for the validity and reliability of all four scales [43].

#### Coping strategies

The fourth tool was the Polish version of Mini-COPE [99], a tool used to measure stress-coping strategies, the original version of which was developed by Carver (1997). The questionnaire includes 28 statements and measures 14 strategies. Due to the factorial structure of Mini-COPE, the fact that that some strategies can be analyzed together has been pointed out [99]. Thus, in our study, they were divided into: (1) active strategies (active coping, planning, positive reframing, seeking instrumental support; Cronbach α = 0.79), (2) emotion-centered strategies (seeking emotional support, acceptance, sense of humor, turning to religion, denial; Cronbach α = 0.63), and (3) avoidance strategies (venting, self-distraction, behavioural disengagement, substance use, self-blame; Cronbach α = 0.73).

#### Rest

In order to analyze the selected health behaviours of academic teachers, the authors designed a short questionnaire, which included three semi-open-ended questions concerning the total daily duration of sleep (number of hours and minutes) and the number of days devoted to rest out of the 36 days of annual vacation leave that each is entitled to. The questions were based on recommendations for adults between 25 and 64 years of age (7–9 hours of sleep) proposed by the National Sleep Foundation (2015), the World Health Organization (2018) and the European Agency for Safety and Health at Work (2020).

#### Data analysis

The main aim of the study was to determine whether workers’ health-promoting behaviours can significantly moderate this relationship. The authors introduced five moderators into statistical analysis, three of which concerned stress-coping strategies (problem-focus PF, emotion-focus EF, and avoidance-focus AF) and two which concerned rest (amount of sleep and rest during vacation leave). Descriptive statistics of independent variables, dependent variables and moderators are presented in supplementary materials in S1 Table.

The first step was to determine the levels of occupational burnout in groups distinguished based on the combination of levels of three moderators: active coping strategies, emotion-centered strategies, and avoidance strategies. Due to the fact that the use of the particular strategies often depends on a number of situational factors and their use is not binary, the respondents were divided into groups based on their score for each type of strategies. For each type of strategy, respondents were divided into those who used them more and less often (i.e. frequent/infrequent use of active strategies) respectively, thus creating segments characterized by unique combinations of stress-coping strategies. Such a division allowed us to obtain eight segments numbering from 13 to 90 respondents. The authors used ANOVA in the analysis of the significance of differences between the segments in terms of occupational burnout and chronic fatigue.

The next step in the analysis was the confirmation of the moderating role of selected health-promoting behaviours. The analysis was conducted independently for each pair of dependent and independent variables (and their components) including each of the five moderators. The authors decided to apply Hayes approach [100] as offering increase in power compared to Baron and Kenny’s approach.

We first checked whether the IV significantly predicts the DV and whether the IV significantly predicts the mediator. Subsequently, by means of complex regression, we checked whether the DV is predicted by the IV and mediating variable.

Basic statistics, correlations, ANOVA and entry regressions were performed using SPSS ver. 27, while we decided on the PROCESS ver. 3.4 procedure with the use of SPSS for the compact results of mediations in the table. This gave us the additional value of Johnson-Neyman’s range of moderation effect [100]. Data set is available in S1 Database in S1 File.

## Results

The sample was comprised of 340 academic teachers, including 16.76% with an MA degree, 52.65% with a doctoral degree, 22.94% with a habilitation degree, and 7.65% with a professorship. 41% of the respondents were male and 59% were female. 22% of respondents had work experience at university up to five years, 19.4% of them 6–10 years, 11–20 years had 32% of the sample, 21–30 years had 15.8% and over 30 years had 10.3% of respondents. The average age of participants was 42 years old, with minimum 23 and maximum 78 years old (see appendix S2 Table).

In the conducted analysis, the independent variables were the levels of job content and job context- related stress; it was found that higher levels of stress were associated with the context of the job. The results obtained indicate that workers cope better with hazards resulting from the character of their job and that organizational factors are their main sources of stress. Dependent variables in the analyzed model were occupational burnout (measured in terms of exhaustion (EX) and disengagement (DIS) and chronic fatigue (CF) (measured in terms of fatigue severity, concentration, motivation, and physical activity). In line with the expectations based on the theoretical assumptions presented, independent and dependent variables were found to be strongly correlated. Occupational burnout is correlated with stress caused both by job content factors *r*(336) = .50, *p* < .01 and job context factors *r*(321) = .40, *p* < .01. A similar correlation can be observed between fatigue and job content-related stress *r*(333) = .50, *p* < .01 as well as between fatigue and job context-related stress *r*(318) = .28, *p* < .01.

In the case of occupational burnout, significantly different results were obtained for four pairs of comparisons. It is worth noting that the group with higher results was characterized by the frequent use of avoidance strategies each time. In the case of chronic fatigue, high results were also obtained in each group characterized by frequent use of avoidance strategies (regardless of the frequency of using the other two types of strategies). At the same time, groups of respondents who frequently use active strategies were characterized by low levels of chronic fatigue, except in cases when they also frequently used avoidance strategies. The results obtained suggest that stress-coping strategies (first and foremost avoidance strategies) can act as moderators, changing the relationships between job content and job context-related stress and occupational burnout and chronic fatigue among academic teachers.

The same procedure of verifying the significance of differences using ANOVA were applied to moderators associated with rest. Dividing the respondents into those who sleep less than seven hours per day (419 minutes or less) on average and those who sleep more than seven hours per day (420 minutes or more), as well as into those who devote more than two weeks of their vacation leave to rest (15 days or more) and those who devote less than two weeks of their vacation leave to rest (14 days or less), four groups of between 72 and 95 respondents were created. As in the case of stress-coping strategies, average results on the scale of occupational burnout and chronic fatigue were compared. The analysis showed that if workers do not rest during their annual vacation leave, they suffer from a significantly higher level of occupational burnout, regardless of the amount of sleep they get, especially when compared to the group who do not get much sleep, but rest during their vacation. In the case of chronic fatigue values, there was only one significant difference–between respondents who sleep on average more than seven hours per day, but do not rest, and respondents who sleep less, but rest during their vacation leave. As in the case of occupational burnout, respondents who do not rest during their vacation leave reported higher results here as well.

The ANOVA results showed the need to conduct further analyses in order to confirm the moderating role of selected health-promoting behaviours. In total, 80 moderations were tested, of which 10 models turned out to significantly differentiate the relationship between stress and its consequences. For the remaining 70 regression analyses, the interactions did not account for any significant proportion of variance in dependent variable scores.

Problem-focused coping (PFC) and emotion-focused coping (EFC) did not moderate any of the analyzed relationships; the first hypothesis should therefore be dismissed. Avoidance-focused coping (AFC) significantly strengthened the relationship between job context stress (CxS), job content stress (CtS), and chronic fatigue (CF) (total score), as well as between CtS on the one side and Ex, fatigue severity (FS), and reduced physical activity (RPA) on the other, which confirms the second hypothesis, albeit mainly with regard to chronic fatigue.

The amount of sleep (ST—sleep time) turned out to be an important moderator only in the case of the relationship between CtS and FS which does not confirm the third hypothesis.

In turn, HDR (hours devoted to rest) was found to weaken the relationship between CxS on the one hand and Ex, RPA, and RC (reduced concentration) on the other, as well as between CtS and RC, which confirms the fourth hypothesis. Results of the described moderation models are presented in supplementary materials in S3 Table.

## Discussion

The ongoing changes of higher education and demands facing academics [1,3,4,101] can lead to negative consequences, namely occupational burnout and chronic fatigue [5,50]. Referring to the JD-R model [25,102] and the transactional model of stress [32,33], the paper presented a study of the relationship between those negative consequences of excessive job demands, and the use of health-promoting behaviours such as coping strategies and rest among academic teachers. The results largely supportthe conceptual model proposed by the authors and highlight the importance of selected positive health practices for the prevention of chronic fatigue and burnout of academic teachers. Work-related stress is significantly correlated with burnout and chronic fatigue among respondents. The results obtained confirm the universality of the JD-R model [28] and the key tenets of the concepts of occupational burnout and chronic fatigue, which assume that the level of stress determines the occurrence of both phenomena [44,103]. In the case of the relationship between job context-related stress (i.e. insufficient resources) and chronic fatigue, the correlation coefficient was almost twice as high as in the case of the relationship between job content hazards and chronic fatigue. This confirms the need to distinguish between motivational processes, leading to more efficient use of resources and an increase in engagement [104], and health processes which lead to negative consequences in the area of health and well-being of employees [34]. Results obtained indicate that job demands have a more significant impact on health processes.

An important aim of the study was to determine whether selected health behaviours of academic teachers significantly moderate the relationship between their workload and the negative consequences thereof. Although numerous works indicate that both stress-coping strategies and rest are important predictors of employee wellbeing [81,105,106], the results obtained suggest that the health behaviours of academic teachers protect them from occupational burnout and chronic fatigue only in some cases.

The first hypothesis, stating that using active and emotion-centered strategies weakens the relationship between stress, and occupational burnout and chronic fatigue, was not confirmed–these strategies did not moderate the relationship between stress and its negative consequences. This result is puzzling, especially in the case of active strategies, which are considered to be the most constructive and effective, due to the sense of control over the stressful situation that comes with them, among other things [107]. Nevertheless, in an unstable external environment characterized by changing the rules of determining the most beneficial course of action, not only emotion-centered strategies, but also active strategies, may not be enough. As mentioned in the introduction, these are precisely the conditions of the academic environment in Poland [2], which makes using individual strategies (both problem-solving and emotion-regulating) an insufficient buffer with which to protect Polish academic teachers from chronic fatigue and burnout, especially when job hazards are not balanced with resources provided by the organization or the socio-economic system.

From among the strategies studied, only avoidance strategies turned out to be an important moderator, which verified the second hypothesis stating that using avoidance strategies strengthens the relationship between stress and its negative consequences. Especially in cases where organizational resources are relatively sparse and job demands are high (causing high levels of job context-related stress), using avoidance strategies significantly strengthens the relationship between stress and the negative consequences, which is in line with previous research [108–110]. Avoidance strategies turn out to be maladaptive, as they do not make it possible to confront the sources of stress and the emotions experienced [108], which largely result from the poor sense of control over the stressful situation [107]. This, moreover, does not allow the worker to use the existing resources and to build resilience [111], and thus aggravates the negative consequences of stress. The study suggests that using avoidance as a stress-coping strategy is negatively correlated with motivational processes but shows no correlation with health processes. Avoidance coping strategies are related with disengagement from active dealing with the stressor, as though as it did not exist, so this strategy provide a temporary solution to a stressful situation without effective elimination of the stressor [112]. Using avoidance strategies therefore weakens the motivational process, which can be linked with a poor sense of control over the situation [107]. This, in turn, is associated with an unwillingness to confront the demands, which results in reduced or suppressed activity. What is more, avoidance strategies divert attention not only from the stressor, but also from the emotions it causes, which is why their use is called disengagement coping [113]. The lack of a moderating effect of avoidance strategies on the relationship between stress and occupational burnout and chronic fatigue in terms of health processes could hypothetically be explained in the context of preventing physical and emotional exhaustion–when job demands are high and resources are limited, reducing activity and diverting attention from the emotions accompanying the strain of the job ensures a more limited use of physical and emotional resources. Even though such a strategy does not allow one to solve the problem in the long term, in the short term it allows one to preserve personal resources and protects one from exhaustion [39].

The verification of the third hypothesis concerning the role of the amount of sleep on the relationship between stress and its negative consequences confirms the moderating influence of sleep, but only on the relationship between job content-related stress and the subjective feeling of fatigue. The study did not confirm the moderating influence of sleep on occupational burnout; these results differ from those obtained in a study conducted among American academic teachers, which confirmed this influence [106]. It is worth noting, however, that according to the results of studies conducted among Dutch academic teachers, highly fatigued workers usually sleep less and need to make more effort to complete the same tasks in comparison to less fatigued workers [114]. In the study, the sleep was found to have a significant effect only on chronic fatigue; this may be explained by the fact that only the duration of sleep was taken into account, but not its quality and regularity or bedtime rituals, which are also important in the context of regeneration [81]. It is possible that, in the case of the relationships analyzed, other variables played a bigger role than sleep, thus obscuring its moderating influence.

As expected, the results confirm the fourth hypothesis saying that the number of vacation days devoted to rest moderates the relationship between stress and its negative consequences. Results indicate that the number of days of vacation leave devoted to rest weakens the relationship between stress and chronic fatigue, especially when it comes to reduced concentration, reduced physical activity, and fatigue severity in connection with job context-related stress. Thus, the fewer days of vacation leave devoted to rest, the greater the possibility of academic teachers experiencing chronic fatigue and occupational burnout. The results obtained do not come as a surprise, as many academic teachers spend part of their vacation catching up on publishing papers. Moreover, due to the specificity of the academic calendar, in the period immediately before vacation they often have a great deal of professional duties related to final and degree exams. Resting during vacation leave also significantly moderates the relationship between job content-related stress and fatigue, understood as reduced concentration. Studies conducted among Danish teachers indicate that activities undertaken during vacation leave are very important for the wellbeing of workers and for the regeneration of their vital resources [106,115]. The results also confirmed the moderating influence of rest on the relationship between stress and occupational burnout, understood as the disengagement of academic teachers, which has also been confirmed by other studies [34].

## Conclusions

The analysis of the results obtained indicates the relevance of creating and implementing health-promoting strategies among academic teachers, which would be aimed at both reducing their workload and promoting practices that facilitate the regeneration of personal resources [116], such as practicing sleep hygiene strategies or strategies for better recovery after work. The time devoted to rest (in both the physical and psychological dimensions, related to thinking about work) was found especially important. Monitoring teachers’ workloads (not only the number and schedule of classes, but also the time needed to complete other tasks) could therefore help to rationally divide the work and, for instance, limit the amount of work done immediately before vacation. Polish academic teachers, as with the majority of academic workers, frequently conduct their research in the summer, because during the academic year they have many duties, including administrative ones. It would be beneficial if the authorities of higher education institutions offered support in the form of the rational and balanced division of work throughout the year, as well as showing restraint when it comes to increasing job demands which disturb the daily resting pattern of academic teachers. In terms of personal stress-coping strategies, adequate workshops devoted to the issues of coping with job demands and regenerating resources, promoting the importance of active breaks and resting at work [117] could be offered, or even special workplace health promotion programs should be created. Simply making people aware of stress-coping strategies and their influence on motivational and health processes can increase the sense of self-efficacy, which is an important personal resource.

The conducted study has several strengths. First, accordingly to the research framework adopted by the authors, the consequences of a burdensome work environment were analyzed from two perspectives: psychological, including the analysis of occupational burnout, as well physical health, including the chronic fatigue, which allow together for a comprehensive assessment of the employee’s well-being. The research analyzed multiple variables that helped to integrate a knowledge from higher education management, organizational psychology and occupational health, which led to filling a cognitive gap in the field of academic teachers’ well-being. Multidimensional analysis responds to the challenges posed by researchers of JD-R model [29].

Secondly, an important research contribution of the authors is the inclusion in the proposed model the selected health-promoting behaviours (positive health practices), such as (1) stress-coping strategies, (2) sleep hygiene, and (3) using annual vacation leave, which play role in the process of the regeneration of vital personal resources of workers and reduce the risk of negative consequences of work overload and burnout. This suggests that such health interventions can therefore be used at the universities as elements of personnel policy and health prevention and thus they can become an important component of supporting of the academic staff well-being.

Thirdly, the results of the research show that using positive health practices regularly reduce the level of negative consequences only to a limited extent, while without changing the factors of the context and content of the work environment, these changes will not be sufficient. The importance of an institutional commitment is also highlighted to creating a positive and healthy work environment and the need for creating the university’s health culture.

And finally, our research is the first study in Poland of a wide range of variables related to the academics teachers job requirements, psychological and physical outcomes of work demands and work-related stress, as well as possible ways of coping. The results of the study fit in with reports from other regions of the world, thus confirming the global need to pay attention to the support of the social capital of universities.

The study was cross-sectional. A limitation of it is the use of subjective self-assessment questionnaires, as answers tend to be influenced by the mood of the respondents and their personal traits [118]. The study focused on self-description, without referring to objective measures. Expanding the study by introducing objective measures would, however, require an insight into the data of the academic institutions, and thus the lack of anonymity could also affect the respondents’ honesty. Moreover, as already mentioned, in further studies into sleep and rest, it would be recommended that not only the asserted quantity, but also the quality, of the analyzed health-promoting practice and other health behaviours should be taken into account.

Another limitation is sampling. The sample (N = 340) is sufficient to perform planned analyses [91,92] and the sample follows the structure of population in terms of the type of higher education institution (public/private) and academic degree of the respondents. But authors are aware that the results are valid within our sample and require confirmation in other contexts. To this end, our findings may not be generalizable to non-respondents, or other countries. Definitely further research should be conducted to confirm observed effects and relations. In further research, it would be beneficial to expand the sample size and measurement methods by means of actual numerical data and qualitative analyses, as well as to conduct longitudinal studies and test the levels of chronic fatigue, burnout, and stress in several periods of time characterized by different work intensity. It would be especially important to conduct these studies during vacation leave in order to verify the influence of the mood of the respondents and situational factors on the results and on the effects of the applied coping strategies. It would also be advisable to compare academic teachers from different countries.

## Supporting information

S1 File Data_academics. (SAV)

S1 Table Descriptive statistics of IV, DV and moderators. (DOCX)

S2 Table Participants’ socio-demographic characteristics. (DOCX)

S3 Table Basic and moderated models and the range of moderations (Johnson-Neyman sig). (DOCX)
