# Supplementary material for: Occupational burnout and chronic fatigue in the work of academic teachers–moderating role of selected health behaviours
Source: PLoS One. 2023 Jan 26;18(1):e0280080. doi: 10.1371/journal.pone.0280080 (PMC9879519; doi:10.1371/journal.pone.0280080)
Supplement: S1 Table — (DOCX) [file pone.0280080.s002.docx]

**S2 Table 1. Descriptive statistics of IV, DV and moderators**

|  | |  |  |  |  |  |  |  |  |  |  |
| --- | --- | --- | --- | --- | --- | --- | --- | --- | --- | --- | --- |
| Variable  (Name) | |  | **Occupational burnout (OB)** | **Chronic fatigue (CF)** | **Job content stress (CtS)** | **Job context stress (CxS)** | **Active strategies (PFC)** | **Emotion centered strategies (EFC)** | **Avoidance strategies (AFC)** | **Rest (part of annual leave) (HDR)** | **Sleeping time  (ST)** |
| Valid | |  | 338 | 335 | 340 | 325 | 341 | 337 | 337 | 336 | 341 |
| Missing | |  | 3 | 6 | 1 | 16 | 0 | 4 | 4 | 5 | 0 |
| Mean | |  | 38.26 | 70.88 | 1.62 | 2.05 | 1.94 | 1.27 | 0.81 | 18.88 | 412.92 |
| Mode | |  | 34 | 76 | 1 | 2 | 2 | 1.3 | 0.7 | 20 | 420.00 |
| Std. Deviation | |  | 7.76 | 20.87 | 0.45 | 0.44 | 0.49 | 0.40 | 0.43 | 10.71 | 64.12 |
| Skewness | |  | 0.098 | -0.322 | 0.436 | -0.493 | -0.354 | 0.036 | 0.916 | 0.302 | -0.791 |
| Kurtosis | |  | -0.234 | -0.351 | -0.44 | 0.134 | 0.862 | -0.041 | 1.88 | -1.145 | 5.138 |
| Percentiles | 25 | | 33.00 | 55.00 | 1.25 | 1.86 | 1.63 | 1.00 | 0.50 | 11.00 | 377.14 |
|  | 50 | | 38.00 | 73.00 | 1.60 | 2.06 | 2.00 | 1.30 | 0.80 | 20.00 | 420.00 |
|  | 75 | | 43.00 | 87.00 | 2.00 | 2.33 | 2.25 | 1.50 | 1.00 | 30.00 | 454.29 |
