# Supplementary material for: Occupational burnout and chronic fatigue in the work of academic teachers–moderating role of selected health behaviours
Source: PLoS One. 2023 Jan 26;18(1):e0280080. doi: 10.1371/journal.pone.0280080 (PMC9879519; doi:10.1371/journal.pone.0280080)
Supplement: S2 Table — (DOCX) [file pone.0280080.s003.docx]

**S2 Table 2. Participants’ socio-demographic characteristics.**

| Study variables | | Frequency  n=340 | Valid percent |
| --- | --- | --- | --- |
| Gender | female | 199 | 58.4 |
|  | male | 141 | 41.3 |
|  | total | 340 | 100.0 |
|  | missing data | 0 |  |
| Age | < 35 | 85 | 25.2 |
|  | 35–44 | 136 | 40.4 |
|  | 45–54 | 72 | 21.4 |
|  | 55–64 | 32 | 9.5 |
|  | > 65 | 12 | 3.6 |
|  | total | 337 | 100.0 |
|  | missing data | 3 |  |
| Degree | below PhD | 59 | 17.3 |
|  | PhD | 178 | 52.2 |
|  | assistant professor | 78 | 22.9 |
|  | full professor | 26 | 7.6 |
|  | total | 340 | 100.0 |
|  | missing data | 0 |  |
| Type of university | public | 309 | 90.9 |
|  | non-public | 31 | 9.1 |
|  | total | 340 | 100.0 |
|  | missing data | 0 |  |
| Working experience at the university | up to 5 years | 75 | 22.1 |
|  | 6–10 years | 66 | 19.5 |
|  | 11–20 years | 109 | 32.2 |
|  | 21–30 years | 54 | 15.9 |
|  | above 30 years | 35 | 10.3 |
|  | total | 339 | 100.0 |
|  | missing data | 1 |  |

**S2 Table 3. Sample description - Age – descriptive statistics.**

| Valid | 337 |
| --- | --- |
| Missing data | 3 |
| Mean | 42.07 |
| Median | 41 |
| Std. Deviation | 10.63 |
| Skewness | 0.702 |
| Std. Error of Skewness | 0.133 |
| Kurtosis | 0.243 |
| Std. Error of Kurtosis | 0.265 |
| Minimum | 23 |
| Maximum | 78 |
