# Supplementary material for: Occupational burnout and chronic fatigue in the work of academic teachers–moderating role of selected health behaviours
Source: PLoS One. 2023 Jan 26;18(1):e0280080. doi: 10.1371/journal.pone.0280080 (PMC9879519; doi:10.1371/journal.pone.0280080)
Supplement: S3 Table — (DOCX) [file pone.0280080.s004.docx]

**S2 Table 3. Basic and moderated models and the range of moderations (Johnson-Neyman sig.)**

| Depen-dent variable | Model |  | Coeff | se | t | p | R | R^2^ | F | p | Moderation | | | Johnson-Neyman sig. | | |
| --- | --- | --- | --- | --- | --- | --- | --- | --- | --- | --- | --- | --- | --- | --- | --- | --- |
|  |  |  |  |  |  |  |  |  |  |  | **R^2^-chng** | **F** | **p** | **Value** | **% below** | **% above** |
| CF | Basic | constant | -.004 | .470 | -.076 | .000 | .503 | .253 | 112.655 | >.001 | .011 | 5.540 | .019 | 2.134 | 96.716 | 3.284 |
|  |  | Ct.S. | .502 | .470 | 10.614 | .000 |  |  |  |  |  |  |  |  |  |  |
|  | Mode- rated | constant | .020 | .045 | .449 | .654 | .607 | .369 | 64.497 | >.001 |  |  |  |  |  |  |
|  |  | Ct.S. | .430 | .045 | 9.602 | .000 |  |  |  |  |  |  |  |  |  |  |
|  |  | AFC | .346 | .045 | 7.671 | .000 |  |  |  |  |  |  |  |  |  |  |
|  |  | Int_1 | -.105 | .045 | -2.354 | .019 |  |  |  |  |  |  |  |  |  |  |
| CF | Basic | constant | .039 | .052 | .747 | .455 | .283 | .080 | 27.637 | >.001 | .013 | 5.131 | .024 | 1.005 | 85.938 | 14.063 |
|  |  | Cx.S. | .276 | .053 | 5.257 | .000 |  |  |  |  |  |  |  |  |  |  |
|  | Mode- rated | constant | .045 | .048 | .927 | .355 | .477 | .228 | 31.070 | >.001 |  |  |  |  |  |  |
|  |  | Cx.S. | .224 | .049 | 4.581 | .000 |  |  |  |  |  |  |  |  |  |  |
|  |  | AFC | .376 | .049 | 7.651 | .000 |  |  |  |  |  |  |  |  |  |  |
|  |  | Int_1 | -.095 | .042 | -2.265 | .024 |  |  |  |  |  |  |  |  |  |  |
| Dis | Basic | constant | -.003 | .045 | -.059 | .953 | .555 | .307 | 149.193 | >.001 | .008 | 4.190 | .041 | 2.716 | 97.923 | 2.077 |
|  |  | Ct.S. | .554 | .045 | 12.214 | .000 |  |  |  |  |  |  |  |  |  |  |
|  | Mode- rated | constant | .018 | .044 | .409 | .683 | .617 | .381 | 68.241 | >.001 |  |  |  |  |  |  |
|  |  | Ct.S. | .496 | .044 | 11.210 | .000 |  |  |  |  |  |  |  |  |  |  |
|  |  | AFC | .275 | .045 | 6.150 | .000 |  |  |  |  |  |  |  |  |  |  |
|  |  | Int_1 | -.091 | .044 | 2.047 | .041 |  |  |  |  |  |  |  |  |  |  |
| FS | Basic | constant | -.004 | .046 | -.086 | .931 | .552 | .305 | 145.850 | >.001 | .017 | 9.314 | .002 | 2.165 | 96.716 | 3.284 |
|  |  | Ct.S. | .551 | .046 | 12.077 | .000 |  |  |  |  |  |  |  |  |  |  |
|  | Mode- rated | constant | .026 | .044 | .596 | .113 | .621 | .385 | 69.109 | >.001 |  |  |  |  |  |  |
|  |  | Ct.S. | .496 | .044 | 11.237 | .583 |  |  |  |  |  |  |  |  |  |  |
|  |  | AFC | .274 | .044 | 6.161 | .361 |  |  |  |  |  |  |  |  |  |  |
|  |  | Int_1 | -.135 | .044 | -3.052 | -.048 |  |  |  |  |  |  |  |  |  |  |
| RPA | Basic | constant | -.005 | .050 | -.095 | .924 | .421 | .177 | 71.508 | >.001 | .013 | 5.952 | .015 | 1.557 | 93.713 | 6.287 |
|  |  | Ct.S. | .421 | .050 | 8.456 | .000 |  |  |  |  |  |  |  |  |  |  |
|  | Mode- rated | constant | .021 | .047 | .447 | .115 | .540 | .292 | 45.332 | >.001 |  |  |  |  |  |  |
|  |  | Ct.S. | .350 | .048 | 7.369 | .444 |  |  |  |  |  |  |  |  |  |  |
|  |  | AFC | .340 | .048 | 7.134 | .434 |  |  |  |  |  |  |  |  |  |  |
|  |  | Int_1 | -.115 | .047 | -2.440 | -.022 |  |  |  |  |  |  |  |  |  |  |
| FS | Basic | constant | -.004 | .046 | -.086 | .931 | .552 | .305 | 145.850 | >.001 | .012 | 5.839 | .016 | -2.232 | 1.194 | 98.806 |
|  |  | Ct.S. | .551 | .046 | 12.077 | .000 |  |  |  |  |  |  |  |  |  |  |
|  | Mode- rated | constant | -.009 | .045 | -.201 | .841 | .568 | .322 | 52.463 | >.001 |  |  |  |  |  |  |
|  |  | Ct.S. | .536 | .045 | 11.784 | .000 |  |  |  |  |  |  |  |  |  |  |
|  |  | ST | .082 | .045 | 1.816 | .070 |  |  |  |  |  |  |  |  |  |  |
|  |  | Int_1 | .126 | .052 | 2.416 | .016 |  |  |  |  |  |  |  |  |  |  |
| Dis | Basic | constant | .041 | .050 | .814 | .416 | .411 | .169 | 65.397 | >.001 | .013 | 5.566 | .019 | 1.556 | 85.266 | 14.734 |
|  |  | Cx.S. | .407 | .050 | 8.087 | .000 |  |  |  |  |  |  |  |  |  |  |
|  | Mode- rated | constant | .010 | .049 | .209 | .834 | .495 | .245 | 34.087 | >.001 |  |  |  |  |  |  |
|  |  | Cx.S. | .359 | .049 | 7.305 | .000 |  |  |  |  |  |  |  |  |  |  |
|  |  | HDR | -.272 | .050 | -5.483 | .000 |  |  |  |  |  |  |  |  |  |  |
|  |  | Int_1 | -.113 | .048 | -2.359 | .019 |  |  |  |  |  |  |  |  |  |  |
| RPA | Basic | constant | .031 | .054 | .578 | .564 | .251 | .063 | 21.353 | >.001 | .015 | 5.373 | .021 | .606 | 72.381 | 27.619 |
|  |  | Cx.S. | .250 | .054 | 4.621 | .000 |  |  |  |  |  |  |  |  |  |  |
|  | Mode- rated | constant | .005 | .053 | .091 | .927 | .350 | .122 | 14.432 | >.001 |  |  |  |  |  |  |
|  |  | Cx.S. | .201 | .054 | 3.740 | .000 |  |  |  |  |  |  |  |  |  |  |
|  |  | HDR | -.235 | .054 | -4.364 | .000 |  |  |  |  |  |  |  |  |  |  |
|  |  | Int_1 | -.121 | .052 | -2.318 | .021 |  |  |  |  |  |  |  |  |  |  |
| RC | Basic | constant | -.005 | .050 | -.093 | .926 | .414 | .171 | 68.568 | >.001 | .010 | 3.915 | .049 | 1.478 | 83.333 | 16.667 |
|  |  | Ct.S. | .414 | .050 | 8.281 | .000 |  |  |  |  |  |  |  |  |  |  |
|  | Mode- rated | constant | -.039 | .053 | -.748 | .455 | .439 | .193 | 25.905 | >.001 |  |  |  |  |  |  |
|  |  | Ct.S. | .350 | .053 | 6.546 | .000 |  |  |  |  |  |  |  |  |  |  |
|  |  | HDR | -.156 | .055 | -2.861 | .004 |  |  |  |  |  |  |  |  |  |  |
|  |  | Int_1 | -.103 | .052 | -1.979 | .049 |  |  |  |  |  |  |  |  |  |  |
| RC | Basic | constant | .022 | .054 | .415 | .679 | .233 | .054 | 18.261 | >.001 | .020 | 7.060 | .008 | .465 | 72.831 | 27.619 |
|  |  | Cx.S. | .232 | .054 | 4.268 | .000 |  |  |  |  |  |  |  |  |  |  |
|  | Mode- rated | constant | -.006 | .054 | -.103 | .918 | .337 | .113 | 13.261 | >.001 |  |  |  |  |  |  |
|  |  | Cx.S. | .186 | .054 | 3.435 | .001 |  |  |  |  |  |  |  |  |  |  |
|  |  | HDR | -.224 | .055 | -4.102 | .000 |  |  |  |  |  |  |  |  |  |  |
|  |  | Int_1 | -.140 | .053 | -2.657 | .008 |  |  |  |  |  |  |  |  |  |  |

Explanations of abbreviations in alphabetical order: AFC - Avoidance strategies, CF – chronic fatigue, CtS - Job content stress, JCxS - ob context stress, Dis – disengagement, FS - fatigue severity, HDR - Rest (part of annual leave), PFC - Active strategies, RC - reduced concentration, RPA – reduced physical activity, ST - Sleeping time.
